# How ‘visual’ is the visual cortex? The interactions between the visual cortex and other sensory, motivational and motor systems as enabling factors for visual perception

**DOI:** 10.1098/rstb.2022.0336

**Published:** 2023-09-25

**Authors:** Cyriel M. A. Pennartz, Matthijs N. Oude Lohuis, Umberto Olcese

**Affiliations:** ^1^ Cognitive and Systems Neuroscience Group, Swammerdam Institute for Life Sciences, University of Amsterdam, Science Park 904, 1098XH Amsterdam, The Netherlands; ^2^ Amsterdam Brain and Cognition, University of Amsterdam, Science Park 904, 1098XH Amsterdam, The Netherlands; ^3^ Champalimaud Research, Champalimaud Foundation, 1400-038 Lisbon, Portugal

**Keywords:** visual cortex, multimodal processing, predictive processing

## Abstract

The definition of the visual cortex is primarily based on the evidence that lesions of this area impair visual perception. However, this does not exclude that the visual cortex may process more information than of retinal origin alone, or that other brain structures contribute to vision. Indeed, research across the past decades has shown that non-visual information, such as neural activity related to reward expectation and value, locomotion, working memory and other sensory modalities, can modulate primary visual cortical responses to retinal inputs. Nevertheless, the function of this non-visual information is poorly understood. Here we review recent evidence, coming primarily from studies in rodents, arguing that non-visual and motor effects in visual cortex play a role in visual processing itself, for instance disentangling direct auditory effects on visual cortex from effects of sound-evoked orofacial movement. These findings are placed in a broader framework casting vision in terms of predictive processing under control of frontal, reward- and motor-related systems. In contrast to the prevalent notion that vision is exclusively constructed by the visual cortical system, we propose that visual percepts are generated by a larger network—the extended visual system—spanning other sensory cortices, supramodal areas and frontal systems.

This article is part of the theme issue ‘Decision and control processes in multisensory perception’.

## 1. Introduction

Traditionally, the primary visual cortex (V1, striate cortex) has been defined based on Brodmann's cytoarchitectonic studies [1] and coincides with the area of cortex that receives photic inputs, relayed from the retina via the lateral geniculate nucleus (LGN). Functionally, lesions of this area (or its upstream pathways, from retina to optic radiation) cause impairments or loss of visual perception, as reported verbally and behaviourally by subjects [2]. Alternatively, the presence of retinotopic mapping may be deployed as a criterion to delineate the visual cortex, but this type of organization also pertains to lower order areas (e.g. superior colliculus (SC), LGN) or higher order areas to which other, non-visual functions are attributed (e.g. posterior parietal cortex, PPC). Yet, the canonical notion that the visual cortex is uniquely positioned to process only visual information, acting as a relay station to transmit this to higher order cortical and thalamic areas for decision making and other executive functions, has been challenged by a number of recent developments. A first challenge is posed by the accumulating evidence that non-visual information is also intensively processed within visual cortical areas, including information from other sensory modalities, motor-related, mnemonic and motivational systems in the brain. With ‘processing’, we indicate the general collection of processes involving manipulation, organization, storage and retrieval of information for the purpose of generating behavioural responses or performing cognitive functions based on sensory inputs. A first goal of this paper will be to review this evidence and outline how this non-visual information interacts, through cortical microcircuitry laid out across cortical laminae, with visual processing streams, both in feedforward and feedback projections and by way of lateral intracortical connections. We will pay particular attention to the question of how visual information may be integrated with, and segregated from, non-visual processing.

A second challenge concerns the more theoretical question of what makes the visual cortex actually ‘visual’. A generally accepted postulate in sensory neuroscience holds that sensory modalities in the brain are defined by the ‘labelled lines’ (nervous pathways) that connect peripheral sense organs to brain areas dedicated to processing the corresponding modality [3]. In general, the specificity of the afferent sensory pathways and their peripheral receptors (i.e. labelled lines) to the brain goes uncontested vis-à-vis the alternative hypothesis of ‘pattern coding’. Pattern coding holds that different sensory modalities can be conveyed along the same nervous pathway by way of distinct temporal patterns of activity (e.g. bursting versus irregular firing). Nonetheless, the labelled-lines hypothesis leaves the question unanswered of how a given cortical area would come to identify a particular pattern of afferent action potentials as ‘visual’ or ‘auditory’ [4,5]—if this identification could be linked to one area in the first place. Part of the answer may lie in the nature of the statistics of photic inputs to thalamus and cortex. Here, ‘statistics’ refers to the statistical properties conveyed by photic inputs considered at a mass scale (e.g. across all optic nerve fibres involved in the transfer of retinal information to thalamus and cortex), which can be argued to be different from those conveyed by other sense organs. For instance, spatial correlations in mass retinal output will be structured differently than those in cochlear output to the brain. However, as further discussed below, we argue that this alone will be insufficient to account for the construction of visual percepts by the brain. Using the fundamental distinction between ‘photic inputs’ (derived from the retina) and ‘vision’ (as a perceptual, conscious phenomenon), we ask which information streams need to be integrated or segregated to build stable, spatially extended visual experiences. In addition to information transmitted to visual cortex, we will review evidence on how visual cortical outputs interact with higher, downstream areas in the genesis of vision.

A key concept in this constructive process is predictive processing (PP), holding that the perceiving brain forges best-guess representations (inferences) of the causes of sensory inputs [6–9]. Thus, the brain is not tasked with exactly ‘copying’ features from the external world into perceptual systems, but does a much more constructive job: building an internal, representational model of what is likely causing the inputs relayed to the brain from the sense organs [10–13]. The key mechanism is that brain areas (often placed at a higher position in a sensory hierarchy) generate ‘predictions’ about (or representations of) the causes of particular sensory inputs, which are compared to actual sensory input such that an error can be computed (i.e. the discrepancy between prediction and input). In turn, this error (often placed at a lower position in the hierarchy, and sent to higher areas) serves to improve the inferential predictions generated at higher levels, as well as to steer the modification of synapses, by which the network is trained [10–15]. PP networks have generative capacities as they allow the (re-)generation of images at the input level, from the space of latent representations encoded at higher levels. These capacities are important, for instance, in view of conceptualizing mechanisms for explaining internally driven forms of visual experience (e.g. imagery, dreaming).

Rather than relying on PP strictly within the visual cortical hierarchy, we argue that modally distinct visual representations arise by more widespread interactions with other modalities (most notably audition, touch, proprioception and the vestibular sense), supramodal systems and more frontal systems regulating and planning motor movements, evaluation and executive decisions. This implies a justification to expand the traditional notion of an anatomically delimited ‘visual cortex’ to a much larger network of corticothalamic areas—the ‘extended visual system’, consisting of core components and auxiliary areas—if one seeks to explain the spatial, situational and modally specific aspects of visual experience.

Our line of argumentation will be built up as follows. First, we will describe non-visual influences impinging on the visual cortical system, for instance sound-evoked responses in primary visual cortex. We will review whether and how non-visual sensory, motor or cognitive variables interact with visual processing in V1 and related areas, and consider various functional interpretations of these non-classical influences. Second, we will dig more deeply into visual and non-visual information processing within the microcircuitry of the cortex, with special emphasis on laminar distinctions, and re-examine interactions between these streams of information at this finer scale. Finally, we will zoom out to larger anatomical scales and develop the concept of the extended visual system, starting from PP and several hallmarks of visual perception such as object and scene stability.

## 2. Non-visual effects on the visual cortex: origins and functional relevance

The rodent visual cortex is commonly subdivided into the primary visual cortex (V1) and a set of secondary visual areas generally referred to as higher order visual areas [16–18]. These areas, similarly to V1, are retinotopically organized [19,20] and play a significant role in processing visual information [21–23]. However, they have also long been shown to process other sensory modalities [24–28] and be involved in, for example, short-term memory, evidence accumulation and decision making [29–33]. For this reason, §§2 and 3 of this review will focus on V1, an area that, in contrast, is classically considered to be uniquely dedicated to visual processing.

### (a) Auditory-evoked responses in primary visual cortex

We will first review the factors that, besides visual stimuli, have recently been shown or hypothesized to evoke or modulate the activity of V1 neurons. First, we will discuss how signals from sensory modalities other than vision are represented in V1. Among these, auditory-evoked responses in particular have been used as a model to unravel the complex nature of non-visual responses in primary visual cortex.

**Figure 1.**
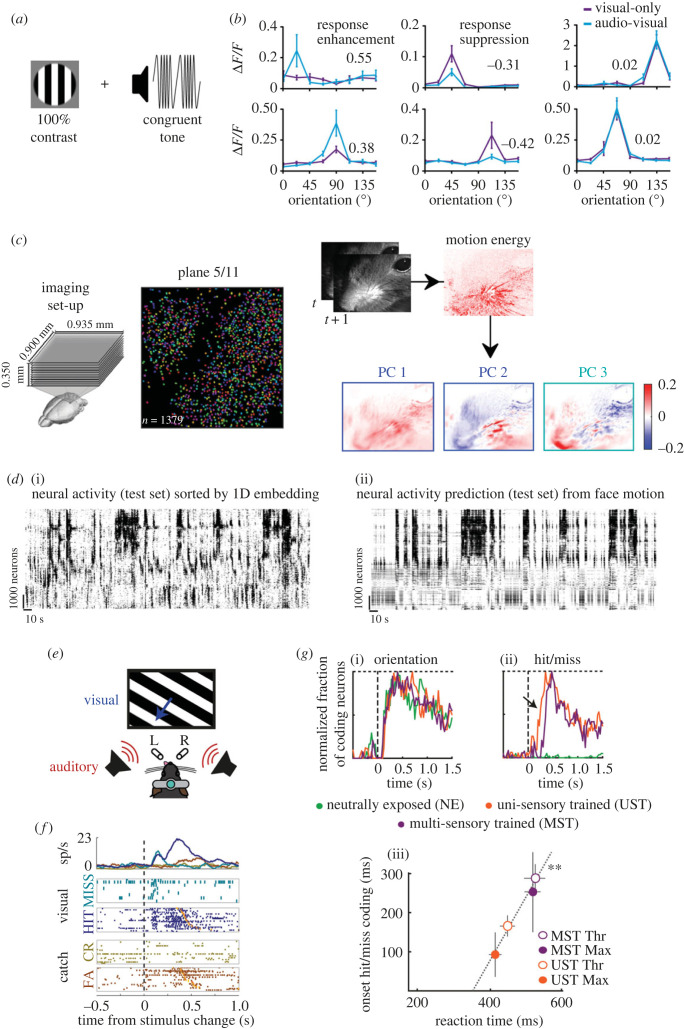
Non-visual responses in primary visual cortex. (*a*) Visual gratings were presented to head-fixed awake mice with or without simultaneous auditory stimuli whose frequency modulation was congruent with the temporal frequency of the gratings. (*b*) Activity of individual V1 neurons was measured via two-photon calcium imaging and revealed a diversity of effects of auditory modulation of V1 activity: enhancement of visual responses (left), suppression (centre) or no effect (right). Panels (*a,b*) have been modified with permission from Meijer *et al.* [39]. (*c*) Simultaneous two-photon calcium imaging (left two panels) and video monitoring of orofacial movements were performed to study whether motion correlates are present in V1 of head-fixed awake mice. Motion energy was extracted from video recordings and dimensionality reduction was performed by principal component (PC) analysis. (*d*) (i) Raster plot of neuronal activity obtained from two-photon calcium imaging, where neurons are ordered following a manifold embedding algorithm that sorts single cells based on how correlated their activity is. (ii) A reduced rank regression approach was used to predict neuronal activity from orofacial motion data. This approach was able to reproduce the experimentally observed patterns of activity in V1, thus indicating how V1 activity encodes motion patterns. Panels (*c,d*) have been modified with permission from Kaplan & Zimmer [40]. (*e*) Head-fixed mice were trained to perform an audiovisual change detection task, in which they had to lick left/right based on whether they detected a change in auditory or visual stimuli, respectively. (*f*) Example single-neuron activity traces (peristimulus time histogram and raster plots) showing different responses to the same visual stimulus based on whether a mouse detected it or not (hit or miss, respectively). FA, false alarm; CR, correct rejection. (*g*) Fraction of neurons significantly encoding either visual orientation (i) or behavioural report (ii) as a function of time after stimulus onset for different task contingencies: neutrally exposed (naive—NE) mice, animals trained to only respond to changes in visual stimuli (UST) and animals trained to respond to changes to both visual and auditory stimuli—see also (*e*). (iii) The earliest moment after stimulus onset at which behavioural report (Hit or Miss) is encoded in V1 neurons varies, for the same visual stimulus as a function of task contingency, with subjectively more complex settings associated with later onsets (see also (ii)), which are tightly correlated with, and precede, reaction time. Panels (*e–g*) have been modified with permission from Oude Lohuis *et al.* [41].

Auditory-evoked responses in V1 have long been observed [34,35]. However, it is only in the last decade that, thanks to the growing availability of techniques for microcircuit dissection in mice, a number of studies revealed the origin and possible effect of such responses on ongoing visual or multi-sensory processing [25,36]. In mice, auditory influences on V1 have been shown to originate mainly from direct projections from auditory cortex, and result in both inhibition and enhancement of activity in V1 [37–39] (figure 1*a,b*). The sign of auditory modulations depends on sound intensity, on whether simultaneous visual stimuli are present or absent [42] and on whether they are temporally congruent or incongruent [39]. While auditory cortical projections to V1 can thus generate sound-evoked responses, these have been mainly interpreted as signals modulating visual processing rather than as intrinsically *auditory* responses. Auditory influences on V1 have also been shown to modulate responses to behaviourally relevant visual stimuli, by a mechanism relying on experience-dependent plasticity [43]. Most studies discussed so far stressed that auditory-evoked responses in V1 mainly reflect global auditory features such as loud onsets [37,42]. However, it was recently shown that audiovisual stimuli elicit auditory and multi-sensory responses in V1 tuned to single auditory features such as pitch [44]. This suggested that V1, by virtue of representing auditory features, might itself contribute to auditory processing, thus disputing the long-standing view of a strict segregation between cortical areas tasked with processing distinct sensory modalities [36].

Nevertheless, new studies have challenged this picture. In fact, most auditory-related activity in V1 might reflect auditory-induced motor reactions—and therefore result from corollary discharge (or its proprioceptive consequences)—rather than actually coding auditory information [45,46]. Specifically, stereotyped motor reactions to distinct auditory stimuli evoke large increases in firing activity in V1, differentially encoding distinct motor responses [47] (figure 1*c,d*). This motor-related activity likely underlies a significant fraction of what was thought to be the consequence of direct auditory projections to V1 [45,46,48]. Significantly, even some features of auditory modulation of visual processing are in line with a potential motor origin. For example, head motion either excites or inhibits V1 activity based on whether it occurs under light or dark conditions, respectively [49,50]; this is analogous to what has been shown for auditory modulation of V1 activity [42]. A warning is warranted, however, against attributing a motor origin to all sound-evoked responses in V1. In fact, monosynaptic connections between auditory cortex and V1 have long been established [25,37,38,42], and in a multi-sensory change detection task, early-onset responses in V1 were found to directly stem from the activity of auditory cortex on top of a large motor-related component [46].

Looking back, which of the earlier results on auditory-evoked activity in V1 actually reflect the effect of direct auditory cortical projections? And what is the function of such auditory signals in V1? In view of the recent developments, these are questions that have to be addressed anew, taking care to disambiguate neural activity that truly has an auditory origin, from that correlating to other phenomena [51]. For example, the function of these auditory signals in the visual system is unclear. Inactivation of V1 was shown not to affect auditory detection (whereas it did impair visual detection), and evidence about whether auditory signals might aid visual processing is mixed (cf. [48] and [46]). Another question which so far received less attention is whether sensory modalities other than audition similarly modulate or are represented in V1. Somatosensory and proprioceptive stimuli have been reported to exert modulatory influences on V1 [37,49,52–54], but data are scarcer for other modalities such as taste and smell. As regards gustatory effects, visual responses in higher order visual cortices were reported to be modulated by behaviourally relevant features associated with visual stimuli such as aversive or appetitive taste [55].

### (b) Non-sensory representations in V1

The previous paragraphs already introduced how the activity in V1 is not only determined by visual information, but is also influenced by other sensory modalities and by non-sensory factors such as body movement.

Several other non-sensory sources of information also exert an influence on V1 by modulating visual responses. This is the case, for instance, for arousal [56–58], locomotion [59–63], attention [62,64–66] and context-dependent modulation [41,62,64–69]. With ‘context’, we mean factors that influence how information about single-sensory stimuli is interpreted. For instance, task rules or the likelihood (expectation) that a certain stimulus occurs can be considered to be contextual factors. In addition, reward-related modulation of visual processing has been documented in V1 and other visual areas [55,70–72]. V1 displays neural correlates of reward expectancy and reward, both on short time scales and periods involving long-term plasticity [73–75]. All of these modulatory influences are generally low dimensional [72], that is, they can be represented in terms of a few modes of activity similarly affecting all neurons in a given area. Moreover, these influences exert long-lasting, tonic effects on the activity of V1 (i.e. lasting for as long as a given contextual factor is present, e.g. seconds or more in the case of behavioural state or locomotion [57,61]). This is visible, for example, in terms of depolarizations or (de)synchronization of firing patterns that are homogeneous across cell populations [55,57,59,64].

In contrast to these modulatory influences, other forms of non-sensory activity appear in V1 as phasic, high-amplitude events which encode non-visual information such as spontaneous motor responses (see above) and task-related behavioural correlates. For instance, orofacial movements, including head motion and eye movements, elicit large, multi-dimensional patterns of activity in V1 [45,47,49,50,59,76–79]. These activity patterns encode a detailed representation of ongoing motion, but—at least for correlates of non-instructed movements—surprisingly remain orthogonal to visual processing ([47]—cf. [80]). This is most striking in the context of a sensory detection or localization task. After learning such a task, V1 displays a large wave of activity that comes after an early-latency, sensory-related peak in V1 activity. This ‘second bump’ is understood as report-related activity resulting from recurrent cortical feedback [78,81,82] and is tightly locked to and anticipates the motor response [41,78,81] (figure 1*e–g*). This widespread wave of activity supposedly originates in pre-motor areas [47,77,82,83] and, upon reaching V1, can even exceed early visually evoked responses in amplitude [41,78]. Yet, its role in visual processing remains poorly understood, because it is not causally involved in visual processing *per se* [81], even if its onset determines for how long V1 itself is required for visual decision making [41]. In fact, while several earlier studies suggested that the first tens of milliseconds of V1 sensory-evoked activity were required to enable decision making [84,85], this was recently extended to up to 200 ms, or more precisely until the onset of the late, report-related *bump* in activity [41]. What is encoded in this late wave of activity remains to be fully uncovered, since current studies have been unable to disambiguate pre-motor (preparatory) activity, report-related correlates and reward anticipation as possible sources of its origin.

An intriguing possibility is that this wave of report-related activity is the neuron-level equivalent of the P3b component of electroencephalography evoked-response potentials in human studies, that has been hypothesized as a marker of conscious access [86,87], but remains highly controversial [88,89]. In fact, similarly to the P3b, this wave of activity follows an earlier, perception-independent peak of sensory-evoked activity and only arises when animals report the detection of a visual stimulus. By disentangling the origin of the P3b, what it encodes and its role in sensory processing and perception, further insights on the mechanisms of conscious vision may thus be facilitated.

Another example of a non-visual influence on V1, that is indicative of how visual cortex also encodes cognitive variables, is navigational information. It has been found that the location of animals in an environment can be decoded from the activity of visual cortex [90,91]. Spatial representations in V1 are coordinated with hippocampal activity [90] and enhance visual representations, especially of familiar environments [91]. However, it cannot yet be excluded that navigational representations in V1 reflect behavioural, cognitive or sensorimotor covariates (e.g. posture, stereotyped movements, reward expectancy or local sensory cues [92]) rather than actual location information, similar to what has been reported for auditory-related activity [45,46].

All in all, non-sensory representations not only occur in V1, but may also exceed visually evoked responses in amplitude, as is for example the case for report-related activity [41,46,47,78]. It remains unaddressed what function these signals, which are certainly energetically expensive and thus should provide some advantage from an evolutionary point of view, might play. In view of their being orthogonal to sensory representations [47], it has been proposed they may not directly interact with ongoing sensory processing in V1 (cf. [80]). Instead, it has been hypothesized that such non-visual signals may play a role in associative learning [76], in correcting visual signals for self-motion [40], or in providing feedback for PP [14,40,93–95]. Nevertheless, mechanistic, circuit-level explanations for how these forms of processing might be implemented are currently still lacking. All of this will be further discussed in the next section, in which we will consider the microcircuit-level organization of visual and non-visual processing in V1.

### (c) Generalizability beyond rodent visual cortex: other sensory modalities and species

Most of the experiments discussed so far were performed in the visual cortex of rodents. It is therefore important to assess to what extent the same principles governing non-visual processing in V1 also apply to other sensory modalities and other species. Most research on other sensory modalities has focused on how vision affects auditory processing. Visual effects have been described for the auditory cortex in primates [96,97], ferrets [98,99], as well as rodents [37,46,100–102]. These influences have been generally found to be weaker compared to auditory responses in V1 [46,100], suggesting that there is an asymmetry between the two sensory systems. Analogously, asymmetries have been reported between somatosensory, visual and vestibular areas [37,52,54,103]. For example, tactile stimuli were found to inhibit V1, but visual stimuli induce depolarizations in somatosensory cortex [37]. Moreover, while correlates of orofacial movement have been found in V1, much weaker motor correlates were reported in auditory cortex [46]. Overall, these results suggest that processing of information from non-primary modalities may not follow the same principles across sensory systems.

A related question is whether the same rules apply to different species. As mentioned, sound-evoked responses in V1 have been reported to occur at the neuronal level in species other than rodents, including non-human primates. It remains to be seen, however, whether the same circuit-level implementations discovered in rodents also hold in cats, ferrets and primates, all species in which multi-sensory processing has also been studied [98,99,104–107]. A concern might be that most of the cross-talk between systems described in rodents might stem from the small size of their brains, which could facilitate widespread cross-modal communication between relatively distant areas. However, recent studies in primates reported that spontaneous body movements could explain a large fraction of variance in the activity of cortical neurons, similar to what has been reported in mice [108] albeit with important differences such as the sign of the modulation [109].

Overall, these results indicate that non-sensory information processing and signalling from other sensory modalities may represent a key aspect of the functional architecture of the visual cortex. Nevertheless, the circuit-level rules underlying interactions between different sources of information still need to be uncovered and will be discussed next.

## 3. Microcircuitry of visual cortex and the integration of non-visual and visual processing

As argued above, non-visual signals are conveyed to visual cortex through projections arising from different parts of the brain. In this section, we will discuss the microcircuitry underlying non-visual effects on visual cortex. We focus in particular on the cortical layers of V1 where these different non-visual projections terminate and modulate activity to understand how they intertwine with photic inputs.

Bottom-up input carrying sensory information to V1 predominantly projects to the thalamorecipient cortical layer 4 (L4) and to a lesser extent L6 [16,110]. The canonical microcircuit of the sensory cortex posits that visual information would then propagate to L2/3, which has strong reciprocal connections with L5 [111,112]. L2/3 neurons form intracortical connections within V1 and with other cortical areas, and L5 neurons constitute the main output of the cortex through local and long-range projections: intracortical projections from L5a and subcortical projections from L5b mainly to the brainstem, spinal cord, basal ganglia and midbrain. Excitatory neurons in L6 then send feedback to the dorsolateral geniculate nucleus, among other projections. Cortical layers are thought to serve different computational functions in cortical processing and in the construction of visual representations [111,112]. In the context of PP, neurons in L2/3 are often thought to signal deviations between predicted and actual inputs (cf. [113]), while activity in L5/6 corresponds to internal representations [14,15]. The laminar distribution of non-visual inputs is therefore highly informative of the nature of their interaction with processing information of retinal origin.

**Figure 2.**
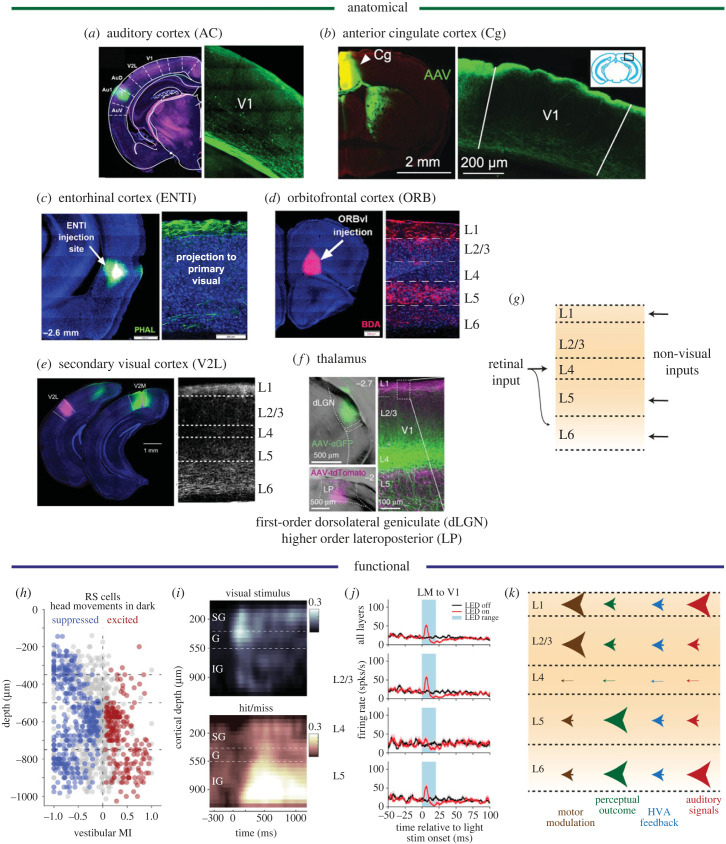
Laminar distribution of non-visual inputs and contextual effects in mouse V1. All anatomical panels come from anterograde tracing studies and show the injection site (left panels) and projection pattern in coronal sections in and around V1 (right panels). (*a*) Left image shows injection site (green) in primary auditory cortex and right panel the projections to V1, from Lohuis *et al.* [46]. Magenta is tdTomato fluorescence. (*b*) Top-down projections from anterior cingulate cortex to V1, from Zhang *et al.* [65]. (*c*) Entorhinal cortex (ENTI) projections to V1 terminate mostly in L1 [115]. (*d*) Ventrolateral orbitofrontal cortex (ORBvl) projections to V1 project to upper L1/2 and L5 [115]. (*e*) Left panel shows injection sites in secondary visual cortex lateral part (V2L, corresponding to LM) and medial part (V2M). Right panel shows projections from V2L to V1 (from V2M not shown), from Young *et al.* [116]. (*f*) Combined tracing of retinal inputs from first-order dorsolateral geniculate (dLGN, green) and higher order LP (magenta) to V1, from Roth *et al.* [110]. (*g*) Summary view of where non-visual axons terminate: mostly in L1 and L5/6. (*h–k*) Functional, electrophysiological evidence. (*h*) Head rotations in the dark modulate V1 excitatory neurons depending on cortical depth. The vestibular modulation index (vMI) quantifies increases (positive vMI) or decreases (negative vMI) in firing rate upon head rotations relative to baseline. Significantly modulated cells are coloured (suppressed, blue; excited, red), from Bouvier *et al.* [49]. (*i*) Sensory and outcome coding are distributed differently across V1 layers. The fraction of neurons significantly coding the presence of a visual stimulus (orientation change) is located more superficially, while neurons coding the perceptual outcome (hit/miss in a detection task) are located in deeper layers, from Oude Lohuis *et al.* [41] (SG: supragranular; G: granular; IG: infragranular layers). (*j*) Optogenetic activation of feedback axons from higher visual cortex (LM) to V1 changes firing in L2/3 and L5, but not L4, from Shen *et al.* [117]. (*k*) Summary view of the laminar distribution of non-visual activity, based on (*h–j*) and references in main text. The strength of perception-related activity in deep layers contrasts with more superficial motor activity and auditory inputs in most superficial and deep hotspots. Arrows denote hotspots of somatic spiking of cells in the respective layers (not the location of anatomical projections as in the upper part of the figure) and the size of the arrows scales with the amount of activity related to the various factors of interest. Note that perceptual outcome and motor modulation are difficult to disentangle and overlap in time (cf. [41,51]).

Several studies using retrograde and anterograde tracing have mapped the projections from non-visual regions to mouse V1 [114] and report strikingly similar laminar profiles (figure 2). Inputs from the auditory cortex—often categorized as ‘lateral’ connections—terminate densely in L1 and L5/6 [38,46,118]. This termination profile is similar to top-down projections from the anterior cingulate cortex involved in attentional control of visual processing [65,115], secondary motor cortex conveying sensorimotor signals [50,94] and entorhinal cortex conveying space- and memory-related signals [115]. Efferents from orbitofrontal cortex, which is generally associated with olfaction, motivation and evaluation of stimuli and actions [119,120], similarly project to L1 and deep layers, but preferentially target L5, upper L2 and lower L1 [115]. These non-visual innervation patterns of V1 also resemble feedback projections from within the visual cortical system, such as from secondary visual area lateromedial (LM) [116,121], and the lateroposterior nucleus (LP) [110], a higher order thalamic region homologous to the pulvinar in primates. Non-visual inputs to V1 thus show a roughly similar distribution of axonal innervations, avoiding L4 and predominantly terminating in L1 and L5/L6, with small differences between these non-visual inputs in the exact distribution within layers and a similarity to local feedback of visual information within the visual system itself (figure 2, upper section) [121–125].

Another major source of non-visual information to V1 is neuromodulatory input. In contrast to the previously discussed non-photic inputs to V1, however, neuromodulatory systems mainly provide a low-dimensional, tonic input to V1 that broadly affects properties such as baseline firing rate or gain [56,58,126–128]. Here we focus on direct sources of non-visual, phasic information to V1 and discuss how these affect visual processing. Therefore, we will not discuss neuromodulatory influences on visual cortex (but see, e.g. [14,57,129–131]).

### (a) Laminar architecture of non-visual signals in V1

Do these similar axonal inputs to V1 also lead to similar modulations across layers of V1? In spite of arriving in the same layers of V1, axonal projections from different source areas might make highly specific connections to neurons with somata in different layers [132]. Axonal projections to L1, for instance, might affect dendrites of neurons in different layers. In fact, in spite of similar axonal termination profiles, non-visual signals appear to modulate V1 activity via different effects on distinct layers.

First, inputs to L1 can either synapse onto dendrites of deeper neurons or recruit the sparse set of interneurons located in L1 itself (for a review on multimodal inputs to L1 see [38]). Indeed, these interneurons respond to motor movements (whisking) as well as auditory noise bursts [133] and may mediate a sound-induced sharpening of visual orientation tuning [38]. Studies that have investigated the cortical depth of different forms of motor-related activity in V1 localize them in both supra- and infragranular layers. Head motion signals can be found in L6 [54] as well as throughout L2/3 and L5, where they both suppress and excite neurons depending on the luminance context [49,50] (figure 2*h*). Locomotion signals predominate in deeper layers [63] and orofacial movements are found in supra- and infragranular layers [46]. Auditory-related activity is more localized in L1 interneurons and upper L2/3 [38,42], with some studies reporting additional hotspots of auditory modulation in deep L5 and L6 [37,46]. The report-related activity seen in Hit trials of detection tasks—which encompasses both perceptual activity related to stimulus detection and motor-related activity related to the reporting—is most prominent in infragranular layers [41,78,82,134] (figure 2*i*). Consistent with these *in vivo* studies on non-visual effects, optogenetic activation of feedback projections from higher visual area LM to V1 and from vibrissal motor cortex (vM1) to primary somatosensory cortex (S1) in cortical slices similarly leads to supragranular and infragranular activity. However, the strongest responses (excitatory postsynaptic potentials) are in L1 interneurons, moderate and variable responses in different cell types in L2/3 and L5, and the weakest effects in L4 [117] (L6 was not investigated; figure 2*j*).

Thus, while canonical processing of photic inputs starts in granular, then supragranular and later in infragranular layers of V1, non-visual signals appear to arrive mostly in superficial and deep layers, thus dominating in extragranular layers. This is consistent with the idea that L4 is mostly driven by feedforward projections from dLGN, while infra- and supragranular layers contextualize the visual stream and associate inputs to it through horizontal and feedback connections [82,111]. Both L1 and L6, and to a lesser extent L2/3 and L5, can be seen as nodes through which convergent inputs from several non-visual brain areas can be integrated with the earliest steps of cortical visual processing [112,135]. The middle layer, L4, is thus wrapped in a ‘contextual sandwich’ by superficial and deep layers. In the context of PP, the pattern displayed in figure 2 is consistent with actual sensory input entering via L4 of V1, while predictions errors may be predominant in L2/3 due to motor feedback and signals from other sensory modalities, whereas (predictive) representations are mostly coded by L5/6 pyramidal cells, in line with the perceptual outcome signals as well as the subcortical outputs from L5/6 cells. Interestingly, a recent study showed how, consistent with predictive coding, feedback from a higher visual area (LM) to V1 pyramidal layer 5 cells was nonlinearly integrated in their apical tuft dendrites [121].

### (b) Interaction of non-visual signals with visual processing

How the activity patterns conveying non-visual information look like in V1 and how they are organized relative to visual activity is key to understanding what they represent and how they are relevant to vision. What can we learn from recent studies about how the integration of non-visual signals with visual processing occurs at the microcircuit level? Do these signals affect the same neurons and how do they shape visual representations?

On the one hand, non-visual activity patterns might be orthogonal to, and independent from, visual processing (see also §2). This could be advantageous in some settings. For example, in a task where the relevant modality (vision or audition) varied across blocks, V1 activity discriminated the block identity of the trials the animal went through [136]. This contextual representation was orthogonal to visual events, such that the contextual representation was maintained across blocks and was not disrupted by visual or auditory stimuli [136]. Also auditory- and motor-related activities have been reported to be orthogonal to visual processing. As introduced in §2, multi-dimensional signals related to ongoing orofacial movements are predominantly orthogonal to visual signals [47]. In an audiovisual detection task where auditory and visual stimuli were explicitly not to be integrated, auditory- and motor-related activity were found in separable neurons and independent of visual responses [46]. Consequently, visual representations were largely unaffected and in audiovisual trials that included task-related movements such as licking, it remained possible to decode features of the presented visual stimulus (i.e. orientation of a moving grating), even in the presence of strong auditory- or motor-related activity.

On the other hand, non-visual signals might selectively affect and interact with visual processing to update representations. For example, locomotion predicts optic flow and specific movements predict detailed visual consequences. Studies on specific visuo-motor interactions do indeed find that they selectively affect visual coding, for example in the case of head or eye movements [49,50], but also locomotion [93,94,137].

Audition relates differently to vision, and auditory stimuli might predict feature-specific or retinotopically specific input based on audiovisual experience. In the context of paired audiovisual stimulation, auditory stimuli were found to affect visual representations (see also §2) [38,43,44]. It may thus be proposed that the specific relationship of the source of non-visual information to visual processing (e.g. auditory to visual and motor to visual) determines the nature of the interaction with visual processing and that different non-visual signals might all prove unique in how they affect V1, given their unique informativeness, anatomical connectivity and predictive relationships to vision. This unique relationship of vision with respect to other modalities has also been postulated to be key to the nature of vision [5]. Sharpening of orientation tuning in V1 has been reported following both auditory stimuli [38] as well as locomotion [59,60,62] and reward [74,138]. It is possible that both locomotion and arousing auditory stimuli affect similar local circuit motifs in V1, and by doing so similarly tune up the gain of the visual system or sharpen visual representations through disinhibition [38,65]. However, it is an open question whether different non-visual inputs tap into similar local circuit motifs—such as L1-mediated control of L2/3 [38,139,140], VIP-mediated disinhibition [65,117,141,142] and L6-mediated gain modulation [54,143]—as only a few studies have investigated the intersection of vision with more than one other modality (e.g. motor, auditory and reward) in the same study.

In sum, the visual cortex expresses the ability to organize non-visual and contextual signals to span orthogonal subspaces such that they do not affect ongoing visual processing, as well as the ability to quickly update visual representations based on relevant non-visual inputs. Whether signals are independent from or directly interact with visual processing is best understood based on the nature of the relationship between vision and the non-visual signal. Contextual signals conveying the current task rule need to be maintained across blocks and should not be disrupted by sensory events, while eye and head movements have specific consequences for visual feature representations and must therefore interact with visual coding neurons. Furthermore, the interaction might be a function of the task being performed. For example, the functional overlap might depend on whether auditory and visual signals need to be integrated [44,144] or segregated [41,46]. Non-visual signals in visual cortex are thus not merely the expression of transient modulatory effects on visual feature representation, but also express ongoing coding of contextual and non-visual events and keep track of larger contextual representations as a network. However, as mentioned before, lesions and inactivation of V1 do cause specific visual deficits, which begs the question which activity or interactions with other systems ultimately determine its visual character. This question will be addressed in the next section.

## 4. Vision as the generative result of large-scale representational brain operations

So far we mainly reviewed how non-visual signals impact on the visual cortex, and how they interact with visual processing through various cortical layers. We will next turn to the more fundamental question of what we actually mean when using the term ‘visual cortex’ given this multiplicity of non-visual signals. First, one may consider the alternative stance that the visual cortex is no longer strictly ‘visual’ given all other inputs, and is therefore better characterized—together with other sensory cortical domains—as ‘multi-sensory cortex’ [36]. However, as visual cortical lesions cause blindness but not deafness (and vice versa for auditory cortical lesions, and so on for other modalities; see also [41] for corresponding optogenetic effects), the specific causal importance of visual cortex for visual perception needs to be accommodated as well. Therefore, it remains justified to denominate Brodmann's areas 17, 18 and 19 in humans (and their homologues in other species) as visual cortex, even though the intensity of cortico-cortical information trafficking with other areas is daunting as compared to the input from LGN or other external afferents (order of magnitude: 100–1000 times heavier [145]). However, the maintenance of the canonical classification of visual cortex does not imply that other brain areas would be irrelevant for vision in a correlative or causal sense.

Etymologically, ‘vision’ stems from the Latin ‘*videre*’ and is thus intimately related to ‘seeing’, meant as (conscious) visual perception. By contrast, terms like ‘visual information’ and ‘visual pathways’ have been connotated not only with visual perception, but more liberally with processing any information of retinal origin, which is more properly labelled as ‘photic signalling’. Direct or indirect retinal pathways to subcortical structures such as the SC, the accessory optic system (AOS), suprachiasmatic nucleus (SCN) and brainstem oculomotor nuclei illustrate the role of photic signalling in e.g. orientation behaviour (SC), optokinetic reflexes and other oculomotor reactions (AOS, brainstem [146]) and day-night rhythmicity (SCN), while not being directly linked to visual perception, as their functioning can proceed in the absence of it. In this sense, ‘visual information’, ‘visual pathways’, etc. if applied to brain systems involved in photically driven functions but not in visual perception, can be dismissed as misnomers.

A second rationale to apply terms like ‘visual information’ with caution is that the very notion of ‘information’ (understood in Shannon's sense [147]) does not strictly permit a specification by sensory modality. Shannon's definition of information is anchored in statistics and hinges on reduction of uncertainty (or ‘surprise’). In this definition, there is no place for different ‘kinds’ of information (e.g. visual), as Shannon information conveys no semantic or content-related meaning (cf. [9,148]).

That it is recommended to refrain from applying ‘visual information’ liberally, is underscored by the (often implicit) assumption that processing such information would be an intrinsic property of the visual cortical system. To see the underlying problem with intrinsic modality-coding, we already referred to the ‘labelled-lines’ hypothesis above. Whereas the anatomical validity of this hypothesis is not at stake here, it does face the problem that the sensory cranial nerves all convey signals (action potentials) of the same type, thus leaving it to the brain to figure out how distinct sensory inputs can be identified as being ‘visual’, ‘auditory’, etc. [4,5]. Thus, cortical cells receiving signals of photic origin have no *intrinsic* way of knowing what these inputs are about. A cell in V1 has no way to figure out where its synaptic inputs are coming from, as it does not possess the knowledge to do so. We previously dubbed this the Modality Identification problem [5]. As already noted above, one way of dealing with this problem is to consider the input of retinal origin not at the level of single-cell spiking, but rather as mass input that collectively conveys certain statistical properties (e.g. spatio-temporal correlation patterns) unique to the visual system as opposed to other modalities having less spatial resolution than vision. As argued in Pennartz [9], however, even statistical regularities in population inputs may be attributed to (i.e. interpreted as belonging to) multiple modalities (think e.g. of neuronal mass dynamics informative about facial images moving across the visual field; from the brain's viewpoint these may relate as well to auditory, somatosensory or other objects). While we are not arguing that sensory input statistics would be irrelevant to specifying sensory modalities, we do argue that other concepts than modally specific input statistics are also important for understanding vision [5]. Specifically, we propose that vision should not be understood as resulting from stand-alone processing of photic signals, but arises as a result of generative computational operations in a brain system that surpasses the (traditional) visual cortical hierarchy in size and complexity.

Our basic proposal is illustrated by the classic finding that luminance/brightness detection tasks can be performed under bilateral striate cortex lesions (thus outside awareness [149,150]), whereas pattern and orientation discrimination tasks do require visual cortex, also in rodents [151]. Given that both tasks are considered ‘simple’, why is this the case? It could be that luminance detection is even simpler than orientation discrimination and can therefore probably rely on subcortical pathways, but this argument leaves unanswered why discriminating oriented gratings does require visual cortex. The most plausible answer, in our view, is that solving an orientation discrimination problem involves not only photic processing *per se*, but also other information such as of motor or proprioceptive origin, namely to compute whether a detected orientation flip is due to an external or internal (self-induced) change, such as a rotation of the head.

Thus, vision is much more complicated than the gradual build-up of cortical response profiles to increasingly more complex visual features along the visual cortical hierarchy. This is furthermore illustrated by the fact that such a system alone does not possess the capacities to stabilize object images in view of eye, head and other body movements. Indeed, the stability of visual representations of our external environment (except for moving objects) is considered a hallmark of conscious experience [152,153]. As reviewed in §§2 and 3, the accumulated evidence demonstrates strong motor-related signalling to the visual cortex, and at least part of the functions of this signalling can be understood as a form of corollary discharge, or motor prediction, that can be used within the visual cortex to correct for body movements to generate stabilized world images, enabling animals to navigate efficiently through the environment (cf. [94]). We may thus assume that visual perception greatly depends on brain systems (especially M1, M2, anterior cingulate cortex [50,94,154]) not canonically considered to be ‘visual cortex’, because lesions in these areas are primarily known to cause motor deficits and affect other sensory systems besides vision as well.

**Figure 3.**
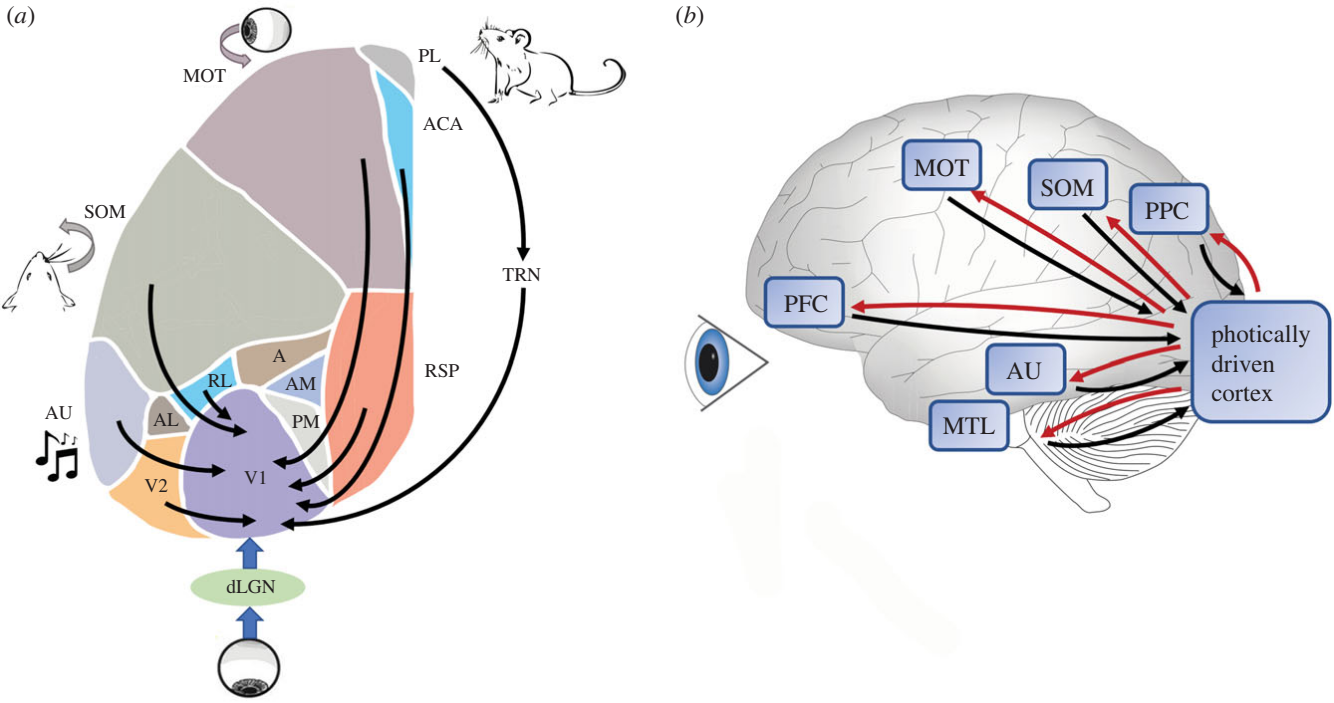
The extended visual cortical system. (*a*) Schematic of the concept of the extended visual system as proposed for mouse neocortex. Across the dorsal surface of mouse neocortex, areas are shown to project to the visual cortical system, in this figure to V1 in particular. Motor areas (MOT) project predictive information to V1, conveying information about expected consequences of eye, head and body movements for vision [50,92,94]. Somatosensory areas (SOM) carry information to V1 on how tactile and proprioceptive information predictably affects visual representations [37,52,53]. Similarly, auditory cortex (AU) transmits sound information to the visual cortex [38,39,42,43]. The anterior cingulate area (ACA) is thought to convey attentional and/or predictive motor signals [65,94], while the prelimbic area (PL) affects visual representations and perceptual decision making via the thalamic reticular nucleus (TRN [155]; RSP, retrosplenial cortex [156]). For the sake of clarity, not all cortical areas projecting to the visual cortex are shown (but see main text: e.g. entorhinal, peri- and postrhinal cortex; orbitofrontal cortex; similarly for thalamic nuclei such as the LP nucleus) and neither is there a set of full connections between higher visual areas and V1 on display. Note that many, but not all, of the connections to V1 have been shown to be reciprocal (if not, higher visual areas are projecting back; e.g. [17]). V2 comprises the subareas P (posterior), LI (laterointermediate) and LM (lateromedial). AL, anterolateral; RL, rostrolateral; A, anterior; AM, anteromedial area; dLGN, dorsal lateral geniculate nucleus; PM, posteromedial area. (*b*) Schematic of the extended visual system as hypothesized for the human brain. ‘Photically driven cortex’ is used here instead of ‘visual cortex’ to emphasize that vision is not intrinsically coded in the occipital lobe, but arises in interaction with other areas. Not all relevant non-visual areas are indicated here; the strength of connections is less well known than for rodent or macaque brains, although the connectivity patterns are generally supported by diffusion tensor imaging, functional connectivity and dynamic studies (e.g. [157,158]). PFC, prefrontal cortex; PPC, posterior parietal cortex; MTL, medial temporal lobe memory system.

Nonetheless, we propose to include these non-visual areas contributing to visual perception in what one may call the ‘extended visual system’ (figure 3). This can be defined both by core components (i.e. classic visual cortical areas, where lesions cause visual but no other deficits) and by satellite components (e.g. motor areas required to generate stable visual representations, however without resulting in blindness when lesioned). Anatomically, the notion of the extended visual system is compatible and in line with studies on the cortical input/output circuitry of V1 and higher visual areas (e.g. [154,159]). How far may the extended visual system stretch out to include other brain areas, classically considered to be non-visual? Much of the answer depends on what the visual system of a given species is tasked to do, and which other capacities enabled by other brain structures may support and enrich vision. The ‘task’ or overall function of the visual system can be broadly construed as informing other brain systems about what is going on in the (visual) world, to subserve subsequent planning, complex decision making and executing goal-directed behaviours (cf. [160]) as opposed to reflexes and automated stimulus-response habits that can be executed without conscious vision [161,162]. However, this characterization should not distract from the fact that visual experience can, in certain cases, become dissociated from overt behaviour, such as in visual imagery during rest and immobility, blindsight [163] and in immobilized but conscious patients (suffering from e.g. paralysis or locked-in syndrome).

To illustrate the question of visual tasking further, let us consider the example of the recognition of camouflaged, partially occluded or other poorly visible objects. Classically, this is approached from the angle of Gestalt psychology, where Gestalt cues such as common motion and contour continuity are used in a bottom-up fashion to achieve figure-ground segregation [164]. However, if the visual field under scrutiny contains a familiar object, memorized knowledge about its contour and textural properties can facilitate its visual detection and perception [9,165,166]. Not only may episodic memory thus support vision, also semantic knowledge can steer our interpretation of, and focal attention to visual images, as illustrated in Jastrow's ambiguous duck-rabbit image [167,168]. In this sense, the widespread recurrent projections from the medial temporal lobe memory system to the visual cortical system form a likely neural substrate for mediating mnemonic influences on visual representations (cf. [16,169]). A similar argument applies to the effects of motivational and emotional memory systems (centred on the amygdala and connected areas such as orbitofrontal cortex) on the visibility, salience and discriminability of stimuli [170,171]. Related to these effects, the influences of reward, reinforcement learning and basic homeostatic drives (e.g. hunger) appear to be sufficiently pervasive [70,172] to affect visual and other sensory microcircuitry directly, e.g. by altering feature tuning properties (cf. [74,75,138,173]) and not only via distal connections (e.g. from frontal to visual cortex).

Also, cortical regions causally linked to non-visual sensory modalities can be considered to form satellite nodes contributing to the extended visual system—especially if changes in these modalities are associated with, or predictive of, changes in visual inputs (cf. [43,44,46,174]). Many examples have been raised to illustrate the impact of e.g. auditory stimuli on visual perception, including the ventriloquist, double-flash and sound-induced collision illusion [175–177] (for somatosensory influences on vision and self-localization, see e.g. [178]). Sections 2 and 3 offer sufficient support to propose the hypothesis that these cross-modal influences on visual perception are at least partly dependent on cortico-cortical interactions, and testing this hypothesis is largely up to future experimental investigations.

Above we already alluded to PP (taken to be wider in scope than classical predictive coding) as an overarching computational framework offering insights into the functioning of the extended visual system. Following the seminal modelling work of e.g. Rao & Ballard [10] and Dayan *et al*. [13] and conceptual schemes for hierarchical Bayesian inference [11,12,179], considerable progress has been recently made in scaling up predictive coding models to multi-layer neural networks that learn and self-organize based on Hebbian plasticity rules [180], multi-sensory networks (combining e.g. the tactile and visual modalities in robots [95]) and networks achieving view-invariant coding of objects [181]. While PP models have traditionally focused on the visual cortical hierarchy [16], we may now begin to discern the contours of PP as a wider explanatory basis for vision in general. Considerable research on the ‘active inference’ variant of PP has been done to show how motor activity giving rise to exploratory movements can be simultaneously conceptualized as giving rise to predictions about the consequences of movement, as reflected in the sensory feedback received upon action execution [182,183]. The incorporation of motor predictions in visual PP is broadly in line with the motor modulation of V1 firing patterns in layer 2–3 of V1 (and to a lesser extent in layer 5–6; figure 2*i,k*), probably originating in part from frontal areas such as M1, M2 and anterior cingulate cortex (figure 2*b*; cf. [50,94]). However, it has been argued that visual and other experiences can proceed in the absence of motricity [161] and thus we propose to also include non-motor, non-visual signals within the notion of the extended visual system.

The rationale to include other sensory modalities in this concept is unitary, but comes in two arguments. First, given the impact of—for example—sound on how we visually perceive, it is unproblematic and even plausible to expand PP models with other, non-visual modalities if these carry predictive power over what inputs of retinal origin may represent (cf. [43,95,152]). Second, cross-modal influences have been argued to be significant in solving the problem of how the brain comes to identify the modality that a particular population pattern of spiking activity belongs to, such that we phenomenologically interpret or perceive this immediately (cf. [5]). This is tied to the ‘consciousness in a bottle’ problem [184], which asks whether a small cortical circuit, kept in a dish for culturing neurons and consisting of two interconnected populations of V1 and MT neurons, could ever generate a percept like ‘motion vision’. It has been argued that V1–MT interactions alone cannot suffice to specify or perceive motion vision, as in this restricted circuitry they cannot be compared to other ongoing streams of non-visual sensory inputs. Consequently, cross-talk between the modalities has been proposed as a basis for a multimodal topology in which each modality occupies a unique position, along with the specific encoded features and input statistics that are unique to that modality.

In summary, a PP-based framework of visual perception is argued to require incorporation not only of motor signalling, but also of non-visual modalities having predictive value for photic inputs. Although space is lacking to expand on the role of other non-motor signals such as bottom-up and top-down attention and various types of memory, these will eventually also need to be included [9,185]. Our conceptualization of the extended visual system does beg the question what the boundaries of this system would be, to prevent commonplace observations such as that, in the brain, everything is (eventually) connected to everything else. This is hard to answer at present and to a significant extent up for future investigations. Tentatively, we may propose that there are likely satellite nodes or adjunct subsystems which are essential for healthy, full-blown vision (such as motor systems for eye and head movement, in the case of mobile subjects), whereas other nodes may be less essential, conditional on learned associations and task complexity (cf. [29,33]), or lacking predictive power when it comes to visual consequences of other sensory events (e.g. airborne odorants are usually invisible but may globally predict visible food items). Thus, boundaries are likely present but rather graded in nature.

It is worthwhile comparing the concept of the extended visual system to other proposals relying on large-scale brain connectivity. Using viral tracing, Zhang *et al*. [154] recently identified a visual network in the mouse brain that consists of the anterior cingulate, retrosplenial and posterior parietal cortices (in addition to visual areas). This network was functionally interpreted as playing a role in top-down control of behaviour and attentional modulation, which differs from our main thesis that higher brain areas such as anterior cingulate and parietal areas are needed for the actual genesis of visual percepts (not merely for their modulation). However, these two interpretations do not contradict each other, as top-down pathways may subserve multiple functions.

The notion of the extended visual system may also seem akin to the Global Neuronal Workspace theory (GNWT) of consciousness, as proposed by Dehaene & Changeux [186] (see also [87,187]). According to GNWT, stimuli reach awareness when they are propagated from early stages of sensory processing to a densely connected, central cortical network where information becomes globally available for broadcasting to many areas subserving executive functions, such as guiding behaviour, working memory and evaluation. In contrast with our current proposal (in line with [9,152]), GNWT is not particularly concerned with phenomenal consciousness but with conscious access, thus focusing on how executive brain systems use stimulus information, rather than on the genesis of visual perception itself. GNWT implies that feedback interactions from higher to lower areas mainly subserve the global availability of information taken as an equivalent of subjective, conscious experience [188], whereas in the current account this feedback serves to convey predictions and other contextual information for constructing visual percepts by way of generative visual computations (cf. [95,179,180,189,190]). Thereby, global availability *per se* is deemed insufficient and underconstrained when it comes to explaining perception. Although various conceptualizations of an ‘extended visual system’ are thus possible, they strongly differ in major theoretical and functional aspects, which calls for deeper, comparative investigations, both empirical and computational.

In conclusion, one may wonder what is to be gained from the notion of ‘the extended visual system’ if one simultaneously retains the conventional denomination of visual cortex based on lesion and anatomical studies. The key advantage, we argue, lies in the shift from an anatomical to a functional focus: instead of relying on a set of brain areas exclusively brought into association with vision (tacitly assuming some kind of intrinsic labelling of the information as being ‘visual’), attention shifts to the question: ‘what else—besides the usual suspects—do we need to build a fully functional visual system?’ For this sort of constructive understanding, as we have argued, PP can serve as a conceptual scaffold, and in turn this scaffold can incorporate motor and non-visual sensory modalities in a more flexible concept of PP that is wide enough to also inform theories of consciousness [8,152,189,191]. While the concept of the extended visual system can be easily applied to other sensory modalities, this applicability does not imply that all of the cortex needs to be classified as ‘multi-sensory’, for instance because some modalities bear stronger correlative or predictive relationships (e.g. somatosensory, vestibular and visual senses) than others.

## Data Availability

This article has no additional data.
